# Bis(pyridine-κ*N*)bis­(triphenyl­phosphine-κ*P*)copper(I) tetra­fluoridoborate

**DOI:** 10.1107/S1600536808008386

**Published:** 2008-04-02

**Authors:** Peter C. Healy

**Affiliations:** a School of Biomolecular and Physical Sciences, Griffith University, Nathan, Brisbane 4111, Australia

## Abstract

The title compound, [Cu(C_5_H_5_N)_2_(C_18_H_15_P)_2_]BF_4_, crystallizes as discrete [(PPh_3_)_2_(py)_2_Cu]^+^ cations and [BF_4_]^−^ anions and is isostructural with the analogous perchlorate salt. The anion is located in close proximity to the pyridine ligands with weak C—H⋯F inter­actions apparent. The P_2_CuN_2_ coordination geometry is pseudo-tetra­hedral, with P—Cu—P and N—Cu—N angles of 116.02 (6) and 101.5 (2)°, respectively.

## Related literature

For background literature on copper(I)–phosphine adducts, see: Hanna *et al.* (1998 ▶, 2005 ▶). For isostructural [(PPh_3_)_2_(py)_2_Cu][ClO_4_], see: Engelhardt *et al.* (1985 ▶)
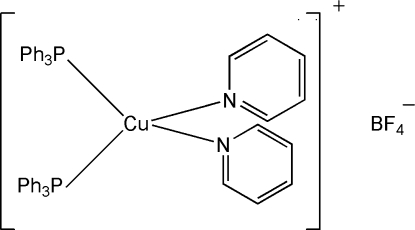


## Experimental

### 

#### Crystal data

[Cu(C_5_H_5_N)_2_(C_18_H_15_P)_2_]BF_4_
*M* *_r_* = 833.10
Triclinic, 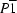

*a* = 10.890 (5) Å
*b* = 13.488 (6) Å
*c* = 15.547 (6) Å
α = 84.97 (3)°
β = 109.59 (3)°
γ = 101.93 (3)°
*V* = 2104.5 (16) Å^3^
*Z* = 2
Mo *K*α radiation
μ = 0.65 mm^−1^
*T* = 295 (2) K
0.50 × 0.30 × 0.15 mm

#### Data collection

Rigaku AFC-7R diffractometer
Absorption correction: ψ scan (North *et al.*, 1968 ▶) *T*_min_ = 0.738, *T*_max_ = 0.909
8222 measured reflections
7393 independent reflections
4745 reflections with *I* > 2σ(*I*)
*R*_int_ = 0.035
3 standard reflections every 150 reflections intensity decay: 0.6%

#### Refinement

*R*[*F* ^2^ > 2σ(*F* ^2^)] = 0.057
*wR*(*F* ^2^) = 0.180
*S* = 1.03
7393 reflections
505 parameters
H-atom parameters constrained
Δρ_max_ = 0.86 e Å^−3^
Δρ_min_ = −0.63 e Å^−3^

### 

Data collection: *MSC/AFC7 Diffractometer Control for Windows* (Molecular Structure Corporation, 1999 ▶); cell refinement: *MSC/AFC7 Diffractometer Control for Windows*; data reduction: *TEXSAN for Windows* (Molecular Structure Corporation, 2001 ▶); program(s) used to solve structure: *TEXSAN for Windows*; program(s) used to refine structure: *TEXSAN for Windows* and *SHELXL97* (Sheldrick, 2008 ▶); molecular graphics: *ORTEP-3* (Farrugia, 1997 ▶); software used to prepare material for publication: *TEXSAN for Windows* and *PLATON* (Spek, 2003 ▶).

## Supplementary Material

Crystal structure: contains datablocks global, I. DOI: 10.1107/S1600536808008386/ng2437sup1.cif

Structure factors: contains datablocks I. DOI: 10.1107/S1600536808008386/ng2437Isup2.hkl

Additional supplementary materials:  crystallographic information; 3D view; checkCIF report

## Figures and Tables

**Table:** 

Cu1—P1	2.2712 (17)
Cu1—P2	2.2955 (16)
Cu1—N1	2.091 (4)
Cu1—N2	2.113 (5)

**Table:** 

P1—Cu1—P2	116.02 (6)
P1—Cu1—N1	112.68 (12)
P1—Cu1—N2	113.03 (13)
P2—Cu1—N1	109.04 (12)
P2—Cu1—N2	103.22 (12)
N1—Cu1—N2	101.51 (17)

**Table 2:** Hydrogen-bond geometry (Å, °)

*D*—H⋯*A*	*D*—H	H⋯*A*	*D*⋯*A*	*D*—H⋯*A*
C11—H11⋯F2	0.95	2.43	3.157 (12)	133
C22—H22⋯F1^i^	0.95	2.53	3.421 (11)	156

Symmetry code: (i) 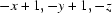.

**Fig. 1.**
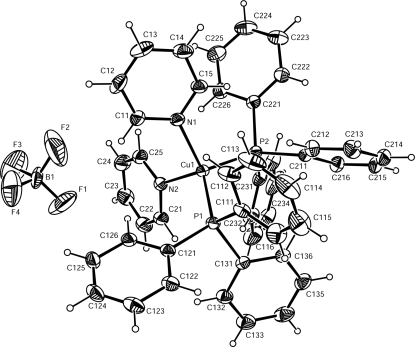
View of the title compound with the atom numbering scheme. Displacement ellipsoids for non-H atoms are drawn at the 30% probability level. H atoms are included with arbitrary radii.

## supplementary crystallographic information

### Comment

The structure of (I) consists of discrete [PPh_3_)_2_(pyr)_2_Cu]^+^ cations and [BF_4_]^-^ anions (Fig. 1) and is isostructural with the analogous perchlorate complex (Engelhardt *et al.*, 1985). The anion is located in the neighbourhood of the pyridine ligands on the cation with weak C—H···F interactions apparent (Table 2). The P_2_CuN_2_ coordination geometry (Table 1) is pseudo-tetrahedral with P—Cu—P and N—Cu—N angles of 116.02 (6) and 101.51 (17)° respectively. For comparison, the P—Cu—P and N—Cu—N angles for [PPh_3_)_2_(CH_3_CN)_2_]BF_4_ are 127.1 (1) and 99.5 (4)° (Hanna *et al.*, 1998). The Cu—P bond lengths of 2.271 (2), 2.296 (2)Å for (I) are similar to the values of 2.269 (1), 2.287 (2)Å recorded for [PPh_3_)_2_(CH_3_CN)_2_]BF_4_. The Cu—N bond lengths for (I), however, are longer with values of 2.091 (4), 2.113 (5)Å compared to 2.039 (4), 2.049 (2) Å. These differences in bond length and angular geometries are likely to a result of increased steric crowding of the 'two dimensional' pyridine ligand by comparison with the linear acetonitrile ligand.

### Experimental

[(PPh_3_)_3_CuBF_4_] (Hanna *et al.*, 2005) (0.52 g, 0.540 mmol) was dissolved in 15 ml boiling ethanol. Pyridine (2 ml) was added and the solution allowed to cool to room temperature. Slow evaporation of solvent resulted in crystallization of colourless crystals of the complex suitable for X-ray diffraction studies.

### Refinement

H atoms were constrained as riding atoms, with C—H set to 0.95 Å. *U*_iso_(H) values were set to 1.2*U*_eq_ of the parent atom.

### Crystal data

**Table:**

[Cu(C_5_H_5_N)_2_(C_18_H_15_P)_2_]BF_4_	*Z* = 2
*M**_r_* = 833.10	*F*_000_ = 860
Triclinic, *P*1	*D*_x_ = 1.315 Mg m^−^^3^
Hall symbol: -P 1	Mo *K*α radiation λ = 0.7107 Å
*a* = 10.890 (5) Å	Cell parameters from 23 reflections
*b* = 13.488 (6) Å	θ = 12.5–14.9º
*c* = 15.547 (6) Å	µ = 0.65 mm^−^^1^
α = 84.97 (3)º	*T* = 295 (2) K
β = 109.59 (3)º	Prismatic, colourless
γ = 101.93 (3)º	0.50 × 0.30 × 0.15 mm
*V* = 2104.5 (16) Å^3^	

### Data collection

**Table:**

Rigaku AFC-7R diffractometer	*R*_int_ = 0.035
Radiation source: Rigaku rotating anode	θ_max_ = 25.0º
Monochromator: graphite	θ_min_ = 2.8º
*T* = 295(2) K	*h* = −12→5
ω–2θ scans	*k* = −15→16
Absorption correction: ψ scan(North *et al.*, 1968)	*l* = −17→18
*T*_min_ = 0.738, *T*_max_ = 0.909	3 standard reflections
8222 measured reflections	every 150 reflections
7393 independent reflections	intensity decay: 0.6%
4745 reflections with *I* > 2σ(*I*)	

### Refinement

**Table:**

Refinement on *F*^2^	Secondary atom site location: difference Fourier map
Least-squares matrix: full	Hydrogen site location: inferred from neighbouring sites
*R*[*F*^2^ > 2σ(*F*^2^)] = 0.057	H-atom parameters constrained
*wR*(*F*^2^) = 0.180	*w* = 1/[σ^2^(*F*_o_^2^) + (0.0978*P*)^2^ + 0.8077*P*] where *P* = (*F*_o_^2^ + 2*F*_c_^2^)/3
*S* = 1.03	(Δ/σ)_max_ = 0.003
7393 reflections	Δρ_max_ = 0.86 e Å^−^^3^
505 parameters	Δρ_min_ = −0.63 e Å^−^^3^
Primary atom site location: structure-invariant direct methods	Extinction correction: none

### Special details

**Table:**

Experimental. The scan width was (1.63 + 0.30tanθ)° with an ω scan speed of 16° per minute (up to 4 scans to achieve I/σ(I) > 10). Stationary background counts were recorded at each end of the scan, and the scan time:background time ratio was 2:1.
Geometry. Bond distances, angles *etc*. have been calculated using the rounded fractional coordinates. All su's are estimated from the variances of the (full) variance-covariance matrix. The cell e.s.d.'s are taken into account in the estimation of distances, angles and torsion angles
Refinement. Refinement on *F*^2^ for ALL reflections except those flagged by the user for potential systematic errors. Weighted *R*-factors *wR* and all goodnesses of fit *S* are based on *F*^2^, conventional *R*-factors *R* are based on *F*, with *F* set to zero for negative *F*^2^. The observed criterion of *F*^2^ > σ(*F*^2^) is used only for calculating -*R*-factor-obs *etc*. and is not relevant to the choice of reflections for refinement. *R*-factors based on *F*^2^ are statistically about twice as large as those based on *F*, and *R*-factors based on ALL data will be even larger.

### Fractional atomic coordinates and isotropic or equivalent isotropic displacement parameters (Å^2^)

**Table:**

	*x*	*y*	*z*	*U*_iso_*/*U*_eq_	
Cu1	0.46141 (5)	0.28505 (4)	0.26070 (3)	0.0484 (2)	
P1	0.29384 (11)	0.36519 (8)	0.25296 (7)	0.0479 (3)	
P2	0.40161 (11)	0.11504 (8)	0.23049 (8)	0.0474 (3)	
N1	0.6167 (4)	0.3073 (3)	0.3847 (2)	0.0552 (12)	
N2	0.5606 (4)	0.3441 (3)	0.1662 (3)	0.0567 (12)	
C11	0.7030 (5)	0.3947 (4)	0.4011 (4)	0.0717 (19)	
C12	0.8051 (6)	0.4137 (5)	0.4833 (5)	0.093 (2)	
C13	0.8198 (6)	0.3400 (7)	0.5494 (4)	0.095 (3)	
C14	0.7324 (7)	0.2507 (5)	0.5330 (4)	0.089 (2)	
C15	0.6334 (5)	0.2366 (4)	0.4502 (3)	0.0636 (17)	
C21	0.4905 (5)	0.3665 (4)	0.0801 (4)	0.0690 (17)	
C22	0.5399 (7)	0.3781 (5)	0.0084 (4)	0.084 (2)	
C23	0.6672 (7)	0.3692 (5)	0.0249 (4)	0.088 (3)	
C24	0.7440 (6)	0.3517 (5)	0.1131 (5)	0.087 (2)	
C25	0.6879 (5)	0.3393 (4)	0.1808 (4)	0.0708 (19)	
C111	0.2321 (5)	0.3414 (4)	0.3501 (3)	0.0587 (17)	
C112	0.3261 (7)	0.3485 (4)	0.4363 (4)	0.081 (2)	
C113	0.2820 (11)	0.3314 (6)	0.5138 (4)	0.109 (4)	
C114	0.1482 (12)	0.3075 (6)	0.5017 (7)	0.116 (4)	
C115	0.0590 (10)	0.2995 (7)	0.4180 (7)	0.120 (4)	
C116	0.0997 (7)	0.3173 (5)	0.3443 (4)	0.092 (3)	
C121	0.3246 (5)	0.5026 (3)	0.2445 (3)	0.0517 (14)	
C122	0.2309 (5)	0.5585 (4)	0.2463 (3)	0.0664 (17)	
C123	0.2574 (7)	0.6627 (4)	0.2386 (4)	0.081 (3)	
C124	0.3758 (7)	0.7121 (4)	0.2304 (4)	0.081 (2)	
C125	0.4710 (6)	0.6597 (4)	0.2285 (4)	0.078 (2)	
C126	0.4445 (5)	0.5546 (4)	0.2352 (3)	0.0624 (17)	
C131	0.1482 (4)	0.3265 (3)	0.1530 (3)	0.0514 (14)	
C132	0.1208 (5)	0.3868 (4)	0.0746 (3)	0.0652 (17)	
C133	0.0207 (6)	0.3505 (5)	−0.0047 (4)	0.083 (2)	
C134	−0.0496 (6)	0.2550 (6)	−0.0061 (4)	0.095 (3)	
C135	−0.0232 (6)	0.1931 (4)	0.0698 (5)	0.086 (2)	
C136	0.0754 (5)	0.2285 (4)	0.1491 (4)	0.0688 (17)	
C211	0.2640 (4)	0.0412 (3)	0.2632 (3)	0.0497 (14)	
C212	0.2424 (5)	0.0666 (4)	0.3404 (4)	0.0647 (17)	
C213	0.1408 (6)	0.0100 (5)	0.3689 (4)	0.081 (2)	
C214	0.0599 (5)	−0.0729 (5)	0.3199 (5)	0.080 (2)	
C215	0.0768 (5)	−0.0978 (4)	0.2436 (5)	0.076 (2)	
C216	0.1779 (5)	−0.0418 (4)	0.2135 (4)	0.0655 (17)	
C221	0.5348 (4)	0.0446 (3)	0.2882 (3)	0.0535 (16)	
C222	0.5237 (5)	−0.0362 (4)	0.3464 (4)	0.0714 (19)	
C223	0.6289 (7)	−0.0850 (5)	0.3898 (5)	0.095 (3)	
C224	0.7473 (7)	−0.0526 (6)	0.3746 (5)	0.099 (3)	
C225	0.7632 (6)	0.0313 (5)	0.3178 (5)	0.089 (3)	
C226	0.6557 (5)	0.0784 (4)	0.2738 (4)	0.073 (2)	
C231	0.3568 (5)	0.0846 (4)	0.1109 (3)	0.0633 (17)	
C232	0.2883 (6)	0.1468 (5)	0.0460 (4)	0.083 (2)	
C233	0.2507 (8)	0.1311 (8)	−0.0457 (5)	0.114 (3)	
C234	0.2762 (8)	0.0461 (11)	−0.0741 (5)	0.142 (5)	
C235	0.3421 (7)	−0.0196 (8)	−0.0116 (6)	0.122 (4)	
C236	0.3837 (5)	−0.0021 (5)	0.0824 (4)	0.081 (2)	
F1	0.7323 (6)	0.5919 (5)	0.1749 (6)	0.242 (4)	
F2	0.8547 (13)	0.5615 (6)	0.2980 (6)	0.353 (7)	
F3	0.9394 (7)	0.6435 (7)	0.2124 (6)	0.240 (5)	
F4	0.8438 (8)	0.7146 (5)	0.2723 (5)	0.213 (4)	
B1	0.8395 (8)	0.6264 (7)	0.2351 (5)	0.098 (3)	
H11	0.69470	0.44550	0.35430	0.0880*	
H12	0.86440	0.47790	0.49230	0.1070*	
H13	0.88830	0.35180	0.60580	0.1140*	
H14	0.73940	0.19850	0.57900	0.1060*	
H15	0.57530	0.17400	0.43990	0.0770*	
H21	0.40050	0.37240	0.06830	0.0820*	
H22	0.48410	0.39180	−0.05140	0.0960*	
H23	0.70380	0.37580	−0.02340	0.1070*	
H24	0.83570	0.34780	0.12670	0.1060*	
H25	0.74110	0.32680	0.24190	0.0870*	
H112	0.41800	0.36370	0.44240	0.0960*	
H113	0.35200	0.33940	0.57290	0.1320*	
H114	0.12670	0.29610	0.55720	0.1500*	
H115	−0.02860	0.28300	0.41460	0.1400*	
H116	0.03420	0.31330	0.28560	0.1120*	
H122	0.14850	0.52430	0.25160	0.0770*	
H123	0.19380	0.70050	0.24120	0.0930*	
H124	0.39260	0.78340	0.22600	0.0970*	
H125	0.55260	0.69330	0.22200	0.0920*	
H126	0.50750	0.51780	0.23380	0.0730*	
H132	0.16970	0.45360	0.07600	0.0780*	
H133	0.00500	0.39260	−0.05840	0.1000*	
H134	−0.11670	0.23070	−0.06050	0.1150*	
H135	−0.07350	0.12650	0.06790	0.1020*	
H136	0.09400	0.18470	0.20230	0.0820*	
H212	0.29830	0.12350	0.37360	0.0760*	
H213	0.12760	0.02840	0.42240	0.0970*	
H214	−0.00790	−0.11300	0.34070	0.1010*	
H215	0.02100	−0.15430	0.20910	0.0950*	
H216	0.18950	−0.06030	0.16000	0.0760*	
H222	0.44130	−0.05930	0.35670	0.0840*	
H223	0.61980	−0.14040	0.43020	0.1160*	
H224	0.81840	−0.08710	0.40250	0.1170*	
H225	0.84740	0.05550	0.31100	0.1070*	
H226	0.66510	0.13490	0.23350	0.0850*	
H232	0.26550	0.20440	0.06510	0.0950*	
H233	0.20620	0.17630	−0.08840	0.1410*	
H234	0.25070	0.03440	−0.13760	0.1820*	
H235	0.36150	−0.07740	−0.03180	0.1450*	
H236	0.42900	−0.04800	0.12590	0.1030*	

### Atomic displacement parameters (Å^2^)

**Table:**

	*U*^11^	*U*^22^	*U*^33^	*U*^12^	*U*^13^	*U*^23^
Cu1	0.0446 (3)	0.0460 (3)	0.0477 (3)	0.0085 (2)	0.0067 (2)	0.0012 (2)
P1	0.0507 (6)	0.0444 (6)	0.0448 (6)	0.0139 (5)	0.0081 (5)	0.0010 (5)
P2	0.0475 (6)	0.0450 (6)	0.0470 (6)	0.0069 (5)	0.0114 (5)	−0.0069 (5)
N1	0.054 (2)	0.052 (2)	0.050 (2)	0.0125 (18)	0.0021 (17)	−0.0040 (17)
N2	0.053 (2)	0.060 (2)	0.054 (2)	0.0083 (18)	0.0175 (18)	0.0096 (18)
C11	0.060 (3)	0.056 (3)	0.079 (4)	0.004 (2)	−0.001 (3)	−0.008 (3)
C12	0.058 (3)	0.088 (4)	0.110 (5)	−0.001 (3)	−0.002 (3)	−0.043 (4)
C13	0.071 (4)	0.141 (6)	0.059 (3)	0.035 (4)	−0.010 (3)	−0.026 (4)
C14	0.094 (4)	0.102 (5)	0.055 (3)	0.030 (4)	0.000 (3)	0.006 (3)
C15	0.068 (3)	0.066 (3)	0.046 (3)	0.016 (3)	0.003 (2)	0.001 (2)
C21	0.063 (3)	0.075 (3)	0.065 (3)	0.016 (3)	0.019 (3)	0.019 (3)
C22	0.093 (4)	0.089 (4)	0.068 (4)	0.020 (3)	0.028 (3)	0.022 (3)
C23	0.099 (5)	0.095 (4)	0.086 (4)	0.021 (4)	0.053 (4)	0.015 (3)
C24	0.063 (3)	0.110 (5)	0.093 (4)	0.020 (3)	0.035 (3)	0.014 (4)
C25	0.060 (3)	0.082 (4)	0.066 (3)	0.012 (3)	0.019 (3)	0.012 (3)
C111	0.077 (3)	0.053 (3)	0.053 (3)	0.025 (2)	0.024 (2)	0.005 (2)
C112	0.117 (5)	0.081 (4)	0.052 (3)	0.049 (4)	0.018 (3)	0.003 (3)
C113	0.185 (9)	0.096 (5)	0.058 (4)	0.074 (6)	0.032 (5)	0.013 (3)
C114	0.185 (9)	0.095 (5)	0.110 (6)	0.055 (6)	0.093 (7)	0.023 (5)
C115	0.145 (7)	0.130 (7)	0.115 (6)	0.024 (6)	0.086 (6)	0.012 (5)
C116	0.099 (5)	0.110 (5)	0.082 (4)	0.025 (4)	0.048 (4)	0.004 (4)
C121	0.059 (3)	0.049 (2)	0.040 (2)	0.014 (2)	0.0051 (19)	0.0008 (18)
C122	0.075 (3)	0.059 (3)	0.063 (3)	0.024 (3)	0.013 (3)	0.000 (2)
C123	0.117 (5)	0.062 (4)	0.068 (4)	0.042 (4)	0.019 (3)	0.000 (3)
C124	0.117 (5)	0.042 (3)	0.068 (3)	0.016 (3)	0.009 (3)	−0.003 (2)
C125	0.085 (4)	0.052 (3)	0.074 (4)	−0.004 (3)	0.007 (3)	0.004 (3)
C126	0.063 (3)	0.057 (3)	0.054 (3)	0.013 (2)	0.001 (2)	0.000 (2)
C131	0.044 (2)	0.056 (3)	0.052 (2)	0.013 (2)	0.0101 (19)	−0.003 (2)
C132	0.071 (3)	0.069 (3)	0.051 (3)	0.019 (3)	0.011 (2)	0.001 (2)
C133	0.091 (4)	0.090 (4)	0.053 (3)	0.025 (3)	−0.001 (3)	−0.006 (3)
C134	0.083 (4)	0.096 (5)	0.082 (4)	0.026 (4)	−0.018 (3)	−0.030 (4)
C135	0.063 (3)	0.067 (4)	0.106 (5)	0.005 (3)	−0.001 (3)	−0.022 (3)
C136	0.055 (3)	0.062 (3)	0.075 (3)	0.011 (2)	0.004 (2)	0.004 (3)
C211	0.047 (2)	0.042 (2)	0.057 (3)	0.0114 (19)	0.012 (2)	0.0024 (19)
C212	0.070 (3)	0.061 (3)	0.065 (3)	0.008 (2)	0.026 (3)	−0.006 (2)
C213	0.089 (4)	0.084 (4)	0.084 (4)	0.022 (3)	0.047 (3)	0.010 (3)
C214	0.058 (3)	0.074 (4)	0.112 (5)	0.013 (3)	0.035 (3)	0.014 (3)
C215	0.055 (3)	0.061 (3)	0.104 (5)	−0.001 (2)	0.021 (3)	−0.001 (3)
C216	0.059 (3)	0.063 (3)	0.072 (3)	0.005 (2)	0.020 (3)	−0.010 (2)
C221	0.053 (3)	0.047 (2)	0.057 (3)	0.012 (2)	0.009 (2)	−0.013 (2)
C222	0.067 (3)	0.064 (3)	0.083 (4)	0.018 (3)	0.022 (3)	0.004 (3)
C223	0.104 (5)	0.094 (5)	0.091 (5)	0.053 (4)	0.022 (4)	0.016 (4)
C224	0.096 (5)	0.126 (6)	0.086 (4)	0.070 (5)	0.018 (4)	0.003 (4)
C225	0.070 (4)	0.109 (5)	0.099 (5)	0.037 (4)	0.029 (3)	−0.008 (4)
C226	0.063 (3)	0.076 (4)	0.087 (4)	0.021 (3)	0.031 (3)	0.000 (3)
C231	0.052 (3)	0.082 (3)	0.053 (3)	0.001 (2)	0.016 (2)	−0.018 (3)
C232	0.072 (4)	0.106 (5)	0.054 (3)	0.003 (3)	0.007 (3)	−0.008 (3)
C233	0.094 (5)	0.173 (8)	0.052 (4)	0.001 (5)	0.006 (3)	−0.005 (4)
C234	0.084 (5)	0.259 (13)	0.059 (4)	−0.031 (7)	0.018 (4)	−0.051 (6)
C235	0.075 (4)	0.187 (9)	0.107 (6)	−0.020 (5)	0.038 (4)	−0.089 (6)
C236	0.066 (3)	0.100 (4)	0.081 (4)	−0.004 (3)	0.031 (3)	−0.038 (3)
F1	0.112 (4)	0.207 (6)	0.317 (9)	0.049 (4)	−0.087 (5)	−0.127 (6)
F2	0.499 (17)	0.195 (7)	0.194 (8)	−0.134 (9)	0.028 (9)	0.070 (6)
F3	0.150 (5)	0.316 (10)	0.282 (9)	−0.008 (6)	0.122 (6)	−0.071 (8)
F4	0.261 (8)	0.156 (5)	0.207 (6)	−0.070 (5)	0.103 (6)	−0.079 (5)
B1	0.081 (5)	0.103 (6)	0.079 (5)	−0.050 (4)	0.025 (4)	0.000 (4)

### Geometric parameters (Å, °)

**Table:**

Cu1—P1	2.2712 (17)	C133—C134	1.353 (10)
Cu1—P2	2.2955 (16)	C134—C135	1.368 (9)
Cu1—N1	2.091 (4)	C135—C136	1.380 (9)
Cu1—N2	2.113 (5)	C211—C216	1.401 (7)
P1—C111	1.828 (5)	C211—C212	1.381 (7)
P1—C121	1.815 (4)	C112—H112	0.9500
P1—C131	1.830 (5)	C212—C213	1.387 (9)
P2—C211	1.817 (5)	C113—H113	0.9800
P2—C221	1.833 (5)	C213—C214	1.378 (9)
P2—C231	1.818 (5)	C114—H114	0.9600
F1—B1	1.255 (12)	C214—C215	1.340 (10)
F2—B1	1.246 (12)	C115—H115	0.9200
F3—B1	1.225 (13)	C215—C216	1.393 (8)
F4—B1	1.353 (11)	C116—H116	0.9500
N1—C15	1.325 (6)	C221—C222	1.366 (7)
N1—C11	1.331 (7)	C221—C226	1.387 (8)
N2—C21	1.344 (7)	C222—C223	1.384 (10)
N2—C25	1.342 (8)	C122—H122	0.9500
C11—C12	1.386 (9)	C223—C224	1.365 (11)
C12—C13	1.359 (10)	C123—H123	0.9500
C13—C14	1.356 (11)	C224—C225	1.392 (10)
C14—C15	1.372 (8)	C124—H124	0.9400
C21—C22	1.376 (9)	C225—C226	1.392 (9)
C22—C23	1.352 (11)	C125—H125	0.9400
C23—C24	1.375 (10)	C126—H126	0.9300
C24—C25	1.366 (9)	C231—C232	1.373 (8)
C111—C116	1.385 (10)	C231—C236	1.403 (8)
C11—H11	0.9500	C132—H132	0.9500
C111—C112	1.384 (8)	C232—C233	1.368 (10)
C112—C113	1.425 (11)	C233—C234	1.374 (16)
C12—H12	0.9600	C133—H133	0.9500
C113—C114	1.377 (18)	C234—C235	1.374 (15)
C13—H13	0.9400	C134—H134	0.9400
C114—C115	1.335 (15)	C135—H135	0.9500
C14—H14	0.9500	C235—C236	1.403 (11)
C15—H15	0.9300	C136—H136	0.9600
C115—C116	1.347 (13)	C212—H212	0.9400
C121—C126	1.396 (8)	C213—H213	0.9500
C21—H21	0.9500	C214—H214	0.9500
C121—C122	1.397 (8)	C215—H215	0.9500
C22—H22	0.9500	C216—H216	0.9400
C122—C123	1.378 (7)	C222—H222	0.9500
C23—H23	0.9500	C223—H223	0.9400
C123—C124	1.365 (11)	C224—H224	0.9400
C124—C125	1.379 (10)	C225—H225	0.9500
C24—H24	0.9600	C226—H226	0.9500
C25—H25	0.9500	C232—H232	0.9600
C125—C126	1.389 (7)	C233—H233	0.9400
C131—C132	1.385 (6)	C234—H234	0.9500
C131—C136	1.389 (7)	C235—H235	0.9500
C132—C133	1.390 (8)	C236—H236	0.9600
			
Cu1···H112	3.3100	C221···H15	2.9200
Cu1···H126	3.0900	C222···H124^xi^	3.0100
Cu1···H212	3.2900	C224···H213^vii^	3.0100
Cu1···H226	3.4400	C225···H234^x^	2.9600
Cu1···H232	3.2000	C226···H236	3.0700
P2···N2	3.457 (5)	C231···H216	2.6300
F1···C25	3.338 (9)	C232···H21	3.0400
F2···C11	3.157 (12)	C234···H135^xii^	2.8800
F3···C123^i^	3.306 (12)	C235···H135^xii^	2.8600
F4···C215^ii^	3.309 (10)	C235···H235^x^	3.0900
F4···C214^ii^	3.274 (10)	C236···H235^x^	3.0900
F1···H22^iii^	2.5300	C236···H216	2.7200
F1···H126	2.8600	H11···F2	2.4300
F2···H13^iv^	2.7500	H11···H126	2.5600
F2···H11	2.4300	H12···C114^v^	2.9400
F3···H133^iii^	2.7600	H13···F2^iv^	2.7500
F3···H123^i^	2.6100	H13···F3^iv^	2.8200
F3···H215^ii^	2.6900	H14···C214^vii^	2.9800
F3···H13^iv^	2.8200	H15···C221	2.9200
F4···H215^ii^	2.7200	H21···C232	3.0400
F4···H214^ii^	2.6200	H21···H232	2.4300
F4···H114^v^	2.5600	H22···F1^iii^	2.5300
N1···N2	3.255 (6)	H25···N1	2.9300
N2···N1	3.255 (6)	H25···C11	2.8900
N2···P2	3.457 (5)	H25···H226	2.5500
N1···H25	2.9300	H112···Cu1	3.3100
N1···H112	2.8600	H112···N1	2.8600
N2···H126	2.8900	H113···C125^v^	3.0400
C11···C25	3.512 (8)	H114···F4^v^	2.5600
C11···F2	3.157 (12)	H116···H136	2.5600
C15···C221	3.519 (7)	H116···C131	2.7200
C21···C232	3.272 (9)	H116···C136	2.7000
C22···C22^iii^	3.538 (10)	H122···C116	3.0600
C25···C11	3.512 (8)	H122···C111	2.9300
C25···F1	3.338 (9)	H123···F3^vi^	2.6100
C11···H25	2.8900	H124···C222^xiii^	3.0100
C116···C136	3.268 (9)	H126···Cu1	3.0900
C21···H232	2.8900	H126···F1	2.8600
C122···C132	3.423 (7)	H126···H11	2.5600
C123···F3^vi^	3.306 (12)	H126···N2	2.8900
C25···H226	2.7900	H132···C121	2.6500
C132···C122	3.423 (7)	H132···C122	2.9200
C136···C211	3.559 (7)	H133···F3^iii^	2.7600
C136···C116	3.268 (9)	H134···C123^ix^	3.0700
C111···H136	3.0400	H135···C234^xii^	2.8800
C211···C136	3.559 (7)	H135···C235^xii^	2.8600
C111···H122	2.9300	H136···C111	3.0400
C212···C222	3.586 (8)	H136···C116	2.9400
C113···H223^vii^	2.9400	H136···C211	2.8400
C114···H12^v^	2.9400	H136···C212	2.8100
C214···F4^viii^	3.274 (10)	H136···H116	2.5600
C215···F4^viii^	3.309 (10)	H212···Cu1	3.2900
C116···H122	3.0600	H213···C224^vii^	3.0100
C116···H136	2.9400	H214···F4^viii^	2.6200
C216···C236	3.441 (9)	H214···H224^vi^	2.4800
C221···C15	3.519 (7)	H215···F3^viii^	2.6900
C121···H132	2.6500	H215···F4^viii^	2.7200
C122···H132	2.9200	H215···H233^xii^	2.5300
C222···C212	3.586 (8)	H216···C231	2.6300
C123···H134^ix^	3.0700	H216···C236	2.7200
C125···H113^v^	3.0400	H222···C211	2.5600
C226···C236	3.483 (8)	H222···C212	2.9500
C131···H232	2.9400	H222···C216	3.0200
C131···H116	2.7200	H223···C113^vii^	2.9400
C232···C21	3.272 (9)	H224···H214^i^	2.4800
C235···C236^x^	3.464 (11)	H226···C25	2.7900
C235···C235^x^	3.269 (12)	H226···H25	2.5500
C136···H232	2.8700	H226···Cu1	3.4400
C236···C235^x^	3.464 (11)	H232···Cu1	3.2000
C236···C216	3.441 (9)	H232···C131	2.9400
C136···H116	2.7000	H232···C136	2.8700
C236···C226	3.483 (8)	H232···C21	2.8900
C211···H222	2.5600	H232···H21	2.4300
C211···H136	2.8400	H233···H215^xii^	2.5300
C212···H222	2.9500	H234···C225^x^	2.9600
C212···H136	2.8100	H235···C235^x^	3.0900
C214···H14^vii^	2.9800	H235···C236^x^	3.0900
C216···H222	3.0200	H236···C221	2.6900
C221···H236	2.6900	H236···C226	3.0700
			
P1—Cu1—P2	116.02 (6)	C112—C113—H113	115.00
P1—Cu1—N1	112.68 (12)	C212—C213—C214	119.9 (6)
P1—Cu1—N2	113.03 (13)	C114—C113—H113	125.00
P2—Cu1—N1	109.04 (12)	C113—C114—H114	115.00
P2—Cu1—N2	103.22 (12)	C115—C114—H114	125.00
N1—Cu1—N2	101.51 (17)	C213—C214—C215	120.4 (6)
Cu1—P1—C111	115.25 (18)	C214—C215—C216	120.7 (6)
Cu1—P1—C121	117.97 (19)	C114—C115—H115	116.00
Cu1—P1—C131	111.42 (15)	C116—C115—H115	124.00
C111—P1—C121	102.0 (2)	C211—C216—C215	120.2 (5)
C111—P1—C131	105.0 (2)	C111—C116—H116	119.00
C121—P1—C131	103.8 (2)	C115—C116—H116	118.00
Cu1—P2—C211	118.50 (15)	C222—C221—C226	118.4 (5)
Cu1—P2—C221	113.17 (14)	P2—C221—C222	124.3 (4)
Cu1—P2—C231	114.85 (18)	P2—C221—C226	117.3 (3)
C211—P2—C221	101.9 (2)	C121—C122—H122	120.00
C211—P2—C231	102.9 (2)	C221—C222—C223	121.7 (6)
C221—P2—C231	103.6 (2)	C123—C122—H122	120.00
Cu1—N1—C11	120.6 (3)	C222—C223—C224	119.9 (6)
Cu1—N1—C15	122.2 (3)	C124—C123—H123	120.00
C11—N1—C15	117.2 (4)	C122—C123—H123	120.00
Cu1—N2—C21	120.0 (4)	C223—C224—C225	119.9 (7)
Cu1—N2—C25	122.5 (4)	C125—C124—H124	120.00
C21—N2—C25	115.8 (5)	C123—C124—H124	119.00
N1—C11—C12	122.5 (5)	C126—C125—H125	120.00
C11—C12—C13	119.1 (6)	C124—C125—H125	122.00
C12—C13—C14	118.6 (6)	C224—C225—C226	119.3 (7)
C13—C14—C15	119.5 (6)	C121—C126—H126	119.00
N1—C15—C14	123.1 (5)	C221—C226—C225	120.8 (5)
N2—C21—C22	123.8 (6)	C125—C126—H126	120.00
C21—C22—C23	118.8 (6)	C232—C231—C236	118.7 (5)
C22—C23—C24	118.9 (6)	P2—C231—C236	122.9 (4)
C23—C24—C25	119.1 (7)	P2—C231—C232	118.4 (4)
N2—C25—C24	123.4 (6)	C133—C132—H132	120.00
P1—C111—C112	116.9 (5)	C231—C232—C233	123.0 (7)
P1—C111—C116	125.4 (4)	C131—C132—H132	119.00
C112—C111—C116	117.7 (5)	C134—C133—H133	121.00
C12—C11—H11	119.00	C132—C133—H133	119.00
N1—C11—H11	119.00	C232—C233—C234	118.5 (8)
C13—C12—H12	121.00	C135—C134—H134	120.00
C11—C12—H12	120.00	C133—C134—H134	119.00
C111—C112—C113	118.6 (7)	C233—C234—C235	120.5 (8)
C12—C13—H13	120.00	C234—C235—C236	120.9 (9)
C112—C113—C114	119.8 (7)	C134—C135—H135	120.00
C14—C13—H13	121.00	C136—C135—H135	120.00
C13—C14—H14	120.00	C231—C236—C235	118.2 (6)
C15—C14—H14	120.00	C131—C136—H136	120.00
C113—C114—C115	120.7 (10)	C135—C136—H136	120.00
C114—C115—C116	119.9 (11)	C211—C212—H212	119.00
C14—C15—H15	118.00	C213—C212—H212	120.00
N1—C15—H15	119.00	C212—C213—H213	120.00
C111—C116—C115	123.2 (7)	C214—C213—H213	120.00
P1—C121—C122	121.9 (4)	C213—C214—H214	120.00
N2—C21—H21	117.00	C215—C214—H214	120.00
C22—C21—H21	119.00	C214—C215—H215	121.00
P1—C121—C126	119.6 (4)	C216—C215—H215	118.00
C122—C121—C126	118.6 (4)	C211—C216—H216	120.00
C121—C122—C123	120.1 (6)	C215—C216—H216	120.00
C21—C22—H22	120.00	C221—C222—H222	119.00
C23—C22—H22	122.00	C223—C222—H222	120.00
C22—C23—H23	121.00	C222—C223—H223	121.00
C122—C123—C124	120.4 (6)	C224—C223—H223	119.00
C24—C23—H23	120.00	C223—C224—H224	120.00
C123—C124—C125	121.3 (5)	C225—C224—H224	120.00
C23—C24—H24	120.00	C224—C225—H225	119.00
C25—C24—H24	121.00	C226—C225—H225	122.00
C124—C125—C126	118.7 (6)	C221—C226—H226	119.00
C24—C25—H25	119.00	C225—C226—H226	120.00
N2—C25—H25	118.00	C231—C232—H232	119.00
C121—C126—C125	121.0 (5)	C233—C232—H232	118.00
P1—C131—C132	121.3 (3)	C232—C233—H233	121.00
P1—C131—C136	119.7 (4)	C234—C233—H233	121.00
C132—C131—C136	118.1 (4)	C233—C234—H234	119.00
C131—C132—C133	120.5 (5)	C235—C234—H234	120.00
C132—C133—C134	120.0 (6)	C234—C235—H235	120.00
C133—C134—C135	120.8 (6)	C236—C235—H235	119.00
C134—C135—C136	119.8 (6)	C231—C236—H236	121.00
C131—C136—C135	120.8 (5)	C235—C236—H236	121.00
P2—C211—C212	119.4 (4)	F1—B1—F2	106.0 (9)
P2—C211—C216	122.8 (4)	F1—B1—F3	116.6 (8)
C212—C211—C216	117.8 (5)	F1—B1—F4	115.8 (8)
C211—C212—C213	120.9 (5)	F2—B1—F3	107.9 (10)
C111—C112—H112	120.00	F2—B1—F4	106.9 (7)
C113—C112—H112	122.00	F3—B1—F4	103.2 (9)
			
P2—Cu1—P1—C111	76.07 (19)	C231—P2—C211—C216	19.4 (5)
P2—Cu1—P1—C121	−163.25 (17)	Cu1—P2—C221—C222	122.9 (4)
P2—Cu1—P1—C131	−43.36 (16)	Cu1—P2—C221—C226	−54.7 (4)
N1—Cu1—P1—C111	−50.6 (2)	C211—P2—C221—C222	−5.4 (5)
N1—Cu1—P1—C121	70.1 (2)	C211—P2—C221—C226	176.9 (4)
N1—Cu1—P1—C131	−170.05 (19)	C231—P2—C221—C222	−112.0 (5)
N2—Cu1—P1—C111	−165.0 (2)	C231—P2—C221—C226	70.3 (4)
N2—Cu1—P1—C121	−44.3 (2)	Cu1—P2—C231—C232	−37.2 (5)
N2—Cu1—P1—C131	75.6 (2)	Cu1—P2—C231—C236	145.9 (4)
P1—Cu1—P2—C211	−33.12 (18)	C211—P2—C231—C232	93.0 (5)
P1—Cu1—P2—C221	−152.24 (16)	C211—P2—C231—C236	−83.9 (5)
P1—Cu1—P2—C231	89.0 (2)	C221—P2—C231—C232	−161.2 (5)
N1—Cu1—P2—C211	95.4 (2)	C221—P2—C231—C236	21.9 (5)
N1—Cu1—P2—C221	−23.8 (2)	Cu1—N1—C11—C12	178.1 (5)
N1—Cu1—P2—C231	−142.5 (2)	C15—N1—C11—C12	−1.4 (8)
N2—Cu1—P2—C211	−157.3 (2)	Cu1—N1—C15—C14	−178.1 (5)
N2—Cu1—P2—C221	83.6 (2)	C11—N1—C15—C14	1.4 (8)
N2—Cu1—P2—C231	−35.2 (2)	Cu1—N2—C21—C22	−161.4 (5)
P1—Cu1—N1—C11	−76.4 (4)	C25—N2—C21—C22	4.0 (8)
P1—Cu1—N1—C15	103.0 (4)	Cu1—N2—C25—C24	162.2 (5)
P2—Cu1—N1—C11	153.2 (4)	C21—N2—C25—C24	−2.8 (8)
P2—Cu1—N1—C15	−27.3 (4)	N1—C11—C12—C13	1.2 (10)
N2—Cu1—N1—C11	44.8 (4)	C11—C12—C13—C14	−1.0 (11)
N2—Cu1—N1—C15	−135.8 (4)	C12—C13—C14—C15	1.0 (11)
P1—Cu1—N2—C21	−42.9 (4)	C13—C14—C15—N1	−1.2 (10)
P1—Cu1—N2—C25	152.8 (4)	N2—C21—C22—C23	−1.8 (9)
P2—Cu1—N2—C21	83.2 (4)	C21—C22—C23—C24	−1.8 (10)
P2—Cu1—N2—C25	−81.1 (4)	C22—C23—C24—C25	2.9 (10)
N1—Cu1—N2—C21	−163.9 (4)	C23—C24—C25—N2	−0.6 (9)
N1—Cu1—N2—C25	31.8 (4)	P2—C211—C212—C213	−178.0 (5)
Cu1—P1—C111—C112	46.7 (5)	C216—C211—C212—C213	1.6 (8)
Cu1—P1—C111—C116	−133.5 (5)	P2—C211—C216—C215	177.8 (4)
C121—P1—C111—C112	−82.3 (5)	C212—C211—C216—C215	−1.9 (8)
C121—P1—C111—C116	97.5 (5)	C211—C212—C213—C214	0.0 (9)
C131—P1—C111—C112	169.7 (4)	C212—C213—C214—C215	−1.6 (10)
C131—P1—C111—C116	−10.5 (6)	C213—C214—C215—C216	1.4 (10)
Cu1—P1—C121—C122	−175.1 (3)	C214—C215—C216—C211	0.4 (9)
Cu1—P1—C121—C126	5.4 (4)	P2—C221—C222—C223	−178.4 (5)
C111—P1—C121—C122	−47.8 (4)	C226—C221—C222—C223	−0.8 (8)
C111—P1—C121—C126	132.7 (4)	P2—C221—C226—C225	177.6 (5)
C131—P1—C121—C122	61.1 (4)	C222—C221—C226—C225	−0.2 (8)
C131—P1—C121—C126	−118.4 (4)	C221—C222—C223—C224	−0.2 (10)
Cu1—P1—C131—C132	−100.7 (4)	C222—C223—C224—C225	2.0 (11)
Cu1—P1—C131—C136	68.7 (4)	C223—C224—C225—C226	−2.9 (11)
C111—P1—C131—C132	133.9 (4)	C224—C225—C226—C221	2.0 (10)
C111—P1—C131—C136	−56.7 (5)	P2—C231—C232—C233	179.5 (6)
C121—P1—C131—C132	27.3 (5)	C236—C231—C232—C233	−3.4 (10)
C121—P1—C131—C136	−163.4 (4)	P2—C231—C236—C235	178.6 (6)
Cu1—P2—C211—C212	−33.0 (5)	C232—C231—C236—C235	1.6 (9)
Cu1—P2—C211—C216	147.4 (4)	C231—C232—C233—C234	3.7 (13)
C221—P2—C211—C212	91.8 (4)	C232—C233—C234—C235	−2.2 (15)
C221—P2—C211—C216	−87.8 (4)	C233—C234—C235—C236	0.6 (15)
C231—P2—C211—C212	−161.0 (4)	C234—C235—C236—C231	−0.3 (12)

Symmetry codes: (i) *x*+1, *y*, *z*; (ii) *x*+1, *y*+1, *z*; (iii) −*x*+1, −*y*+1, −*z*; (iv) −*x*+2, −*y*+1, −*z*+1; (v) −*x*+1, −*y*+1, −*z*+1; (vi) *x*−1, *y*, *z*; (vii) −*x*+1, −*y*, −*z*+1; (viii) *x*−1, *y*−1, *z*; (ix) −*x*, −*y*+1, −*z*; (x) −*x*+1, −*y*, −*z*; (xi) *x*, *y*−1, *z*; (xii) −*x*, −*y*, −*z*; (xiii) *x*, *y*+1, *z*.

### Hydrogen-bond geometry (Å, °)

**Table:**

*D*—H···*A*	*D*—H	H···*A*	*D*···*A*	*D*—H···*A*
C11—H11···F2	0.9500	2.4300	3.157 (12)	133.00
C22—H22···F1^iii^	0.9500	2.5300	3.421 (11)	156.00

Symmetry codes: (iii) −*x*+1, −*y*+1, −*z*.
